# Accelerated coronary calcium burden in breast cancer patients after radiotherapy: a comparison with age and race matched healthy women

**DOI:** 10.1186/s13014-021-01936-w

**Published:** 2021-11-02

**Authors:** Yu-Hsuan Lai, Helen H. W. Chen, Yi-Shan Tsai

**Affiliations:** 1 Department of Radiation Oncology, National Cheng Kung University Hospital, College of Medicine, National Cheng Kung University, Tainan, Taiwan; 2 Department of Oncology, National Cheng Kung University Hospital, College of Medicine, National Cheng Kung University, Tainan, Taiwan; 3 Department of Medical Imaging, National Cheng Kung University Hospital, College of Medicine, National Cheng Kung University, No. 138 Sheng-Li Rd, Tainan, Taiwan; 4 Institute of Clinical Medicine, College of Medicine, National Cheng Kung University, Tainan, Taiwan

**Keywords:** Coronary artery calcium, Breast cancer, CAC percentiles, Adjuvant radiotherapy

## Abstract

**Background:**

Radiotherapy (RT) might lead to atherosclerotic plaque buildup and coronary artery stenosis of breast cancer (BC) survivors, and coronary artery calcium (CAC) might be a sign of preclinical atherosclerosis. This study explores possible determinants affecting the acceleration of CAC burden in BC patients after adjuvant RT.

**Methods:**

Female BC patients receiving adjuvant RT from 2002 to 2010 were included. All patients received noncontrast computed tomography (NCCT) of thorax before and after adjuvant RT. Their CAC burden was compared with healthy controls from the Multi-Ethnic Study of Atherosclerosis (MESA) cohort. The progression of the CAC burden was manifested by the increment of CAC percentiles (%CACinc).

**Results:**

Ninety-four patients, including both left- and right-side BC, were enrolled in this study. From undergoing the first to second NCCT, the %CACinc in BC patients significantly increased rather than non-BC women. In addition, the %CACinc was significantly higher in left-side than right-side BC patients (p < 0.05), and significant differences in most heart outcomes were found between the two groups. Besides, the lower the mean right coronary artery (RCA) dose, the lower the risks of CAC percentiles increase ≥ 50% after adjusting the disease's laterality.

**Conclusions:**

A significantly higher accelerated CAC burden in BC patients than non-BC women represents that BC could affect accelerated CAC. A higher risk of accelerated CAC burden was found in left-side than right-side BC patients after adjuvant RT. A decrease of the mean RCA dose could reduce more than 50% of the risk of accelerated CAC burden in BC patients.

## Background

Multimodality treatment strategies, including radiotherapy (RT), contribute to improve overall survival in breast cancer (BC) patients. This means a growing population of BC survivors would face late treatment-related toxicities, including radiation-induced cardiac toxicity. Tangential field irradiation to the breast or chest wall holds the advantage in reducing radiation exposure to the contralateral breast/chest wall but remains some unwanted irradiation to the heart, especially in left-side BC. Studies have shown that the cumulative incidence of acute coronary events increases by 16.5% per Gray (Gy) of mean heart dose (MHD) [1, 2]. RT cardiotoxicity might occur decades after radiation and earlier in patients with pre-existing risk factors or disease, regardless of chemotherapy [3].

The heart had been thought of a radioresistant organ. However, there is increasing evidence supporting that the heart is actually a dose-limiting organ in RT [4, 5]. The pathogenesis of radiation-induced coronary artery disease is complex and cardiac dysfunction and subsequent heart failure might be due to vascular toxicity and myocardial ischemia [6]. Radiation-induced heart disease includes a series of harmful effects on the heart, from subclinical histopathological findings to obvious clinical diseases. The effect of damaging the heart may be manifested in the pericardium, myocardium, valves, conduction system, or coronary arteries. Earlier studies explore that the coronary arteries are particularly radiosensitive, and the vascular damage might be responsible for long-term radiation-induced ischemic heart disease [7–9]. The effects of local RT to the blood vessels cause pro-inflammatory responses such as increased wall permeability, release of reactive oxygen, inflammatory agents and increased frequency of intraplaque hemorrhage [10], leading to atherosclerotic plaque buildup and coronary artery stenosis. These deposited plaques are often calcified so that coronary artery calcium (CAC) can be served as a sign of preclinical atherosclerosis. The quantification of CAC, absolute CAC scores, has been reported as an important prognostic imaging biomarker for future coronary artery disease (CAD) in asymptomatic individuals [11–13]. There is currently strong evidence that the higher the CAC scores, the higher the risks of acute coronary events [14–17]. An extra benefit of CAC scoring is that the presence and number of calcified plaques in the coronary arteries are determined by a non-invasive tool, a noncontrast computed tomography (NCCT) scan of thorax, thus facilitates its clinical use.

Until today, the clinically valuable dose constraint for the coronary arteries is not yet fully established. Most studies take the MHD as a surrogate parameter in evaluating radiation-associated cardiac toxicity, but whether this parameter can reflect the real condition of coronary arteries remains uncertain. Therefore, higher radiation doses were hypothesized to correlate with the acceleration of calcium deposits in coronary arteries. The dosimetric data of three main coronary arteries from CT-based planning system were analyzed and drew a new concept, the increment of CAC percentiles (%CACinc), to manifest the acceleration of coronary calcium burden before and after adjuvant RT. This study aimed to explore the relationship of accelerated coronary calcium burden and coronary radiation doses in BC patients receiving adjuvant RT. Furthermore, whether the progression of coronary calcium deposits is affected by other cardiotoxic therapeutic agents such as anthracycline, taxane, or trastuzumab, remains debatable. The impact of a variety of treatment and cardiovascular risk factors on the increment of coronary calcium burden was also investigated.

## Materials and methods

### Patients

From January 2002 to September 2010, 94 consecutive female patients aged 45 to 84 years with pathologically diagnosed BC who underwent mastectomy or breast-conserving surgery (BCS) and a complete course of adjuvant RT with a total dose of ≧ 50 Gy at our institution were included. Each patient received two series of NCCT scans of thorax, one before RT and the other at least one year after RT. The patients who received adjuvant RT for locoregional recurrent disease or inoperable tumor or those who received internal mammary nodal irradiation were excluded. The patients with a history of cigarette smoking, CAD, arrhythmia, and stroke before receiving RT were also excluded. The patient data, including age, laterality of tumor, pathological stage, treatment-related profiles (type of surgery, chemotherapy, hormone therapy, targeted therapy), cardiovascular risk factors such as diabetes, hypertension, hyperlipidemia, and body mass index (BMI), were gathered from the hospital medical records. In Taiwan, the Ministry of Health and Welfare defined overweight at BMI ≧ 24 kg/m^2^ and obese at BMI ≧ 27 kg/m^2^. The Tumor, Node, Metastasis (TNM) stage determined previously was revised based on the Seventh Edition of the American Joint Committee on Cancer (AJCC) Stating System [18]. Institutional Review Board approved this retrospective study.

### Non-BC women as a control group

Non-BC women with cardiovascular risk factors such as diabetes, hypertension, and hyperlipidemia without a history of cigarette smoking, CAD, arrhythmia, or stroke before NCCT were included in this study. Finally, 47 non-BC women aged from 45 to 84 years who underwent two series of NCCT scans of thorax with a time interval of at least one year at our institution were collected as a control group. The values of CAC percentiles and %CACinc were calculated as methods mentioned below.

### Radiation therapy dose and techniques

Our treatment plan was designed based on these studies [19–21]. Tangential techniques were used for the chest wall/breast irradiation. Two 180° opposed isocentric tangential fields with half-beam block techniques were set up according to the clinically determined borders. For mastectomy patients, a median dose of 50 Gy in 1.8–2 Gy per fraction was given to the chest wall, with an additional scar boost dose of 10–20 Gy in T4 disease or close/positive surgical margins. For BCS patients, a median dose of 50 Gy in 1.8–2 Gy per fraction was given to the whole breast, followed by a median boost dose of another 10–16 Gy to the tumor bed. Boost field was delivered with appositional field using electrons or intensity-modulated radiotherapy (IMRT) fields using photons. Regional nodal radiation was tailored to the individual patient at the discretion of the treating physicians.

### Contouring of coronary arteries and dosimetry

Since the CT-based planning system was introduced from August 2006 onwards in our institution, dosimetric data were available in 75 patients. The CT-based planning was based on a NCCT scan of thorax before adjuvant RT, with CT-slice thickness of 0.5 cm. The organs at risk (OARs), including the heart, left anterior descending artery (LAD), left circumflex artery (LCX), and right coronary artery (RCA), were contoured for dosimetric calculation according to the guidance of the validated University of Michigan cardiac atlas [22]. Sometimes it was hard to visualize the coronary arteries directly on the NCCT images. Therefore, the delineation of coronary arteries was conducted based on their anatomic courses and inferred by visible landmarks of the heart. Both the LAD and LCX branched from the left main coronary artery (LMCA), and were outlined by the anterior interventricular groove and left atrioventricular groove. The RCA emerged from the aorta and was identified by the right atrioventricular groove and posterior interventricular sulcus. The contouring of OARs was conducted by a 14-year-experienced cardiovascular radiologist. The dose-volume histograms (DVHs) were generated for the planning target volume (PTV) and OARs. Dosimetric comparisons were made between patients with left-side and right-side BCs.

### Determination of absolute coronary artery calcium score (CACinc)

The absolute CAC score was estimated using a non-electrocardiography (ECG)-gated CT scan of thorax [23, 24] and calculated by commercially available calcium scoring software (Aquarius iNtuition software Version 4.4.7, TeraRecon, Inc, San. Mateo, CA, USA). Each patient’s two series of CT scans before and after adjuvant RT were evaluated by the same cardiovascular radiologist to determine the increment of absolute CAC (CACinc) of coronary artery, including LMCA, LAD, LCX, and RCA. The calcified lesions were selected and labeled, and those above a standard threshold of 130 Hounsfield units (HU) were considered candidate calcifications. The scalar given to the peak HU number in the lesion in question was as follows: 1: ≤ 200 HU, 2: 200–299 HU, 3: 300–399 HU, and 4: 400 + HU. The modified Agatston score [14] was calculated by multiplying the area of the calcified lesion by a scalar, and the sum of all these slice-by-slice calculations was taken to generate the total score for a three-dimensional (3D) lesion.

### Age, gender, race/ethnicity specific CAC percentiles by comparison with the MESA cohort

The Multi-Ethnic Study of Atherosclerosis (MESA) study was designed to examine whether a patient had a high CAC score relative to a healthy and asymptomatic participant with the same age, gender, and race/ethnicity [25]. Patients with ages less than 45 years and more than 84 years were excluded from this study because these two age groups were not included in the MESA cohort. The CAC scores of the 94 BC patients aged 45–84 years were compared with those of their age-matched female Asian MESA cohort and then translated into CAC percentiles; for example, the 75th percentile meant that the given value of CAC score was higher than those of 75% healthy populations. The progression of coronary calcium burden was manifested by the %CACinc, defined as the difference of two CAC percentiles before and after adjuvant RT.

$$\% {\text{CACinc}} = {\text{CAC}}\,{\text{percentile}}\,{\text{after}}\,{\text{RT}} - {\text{CAC}}\,{\text{percentile}}\,{\text{before}}\,{\text{RT}}$$

### Statistical analysis

Baseline characteristics were presented as median (first and third quartile) for continuous variables and number (frequency) for categorical variables. P-value of continuous variables was calculated by Mann–Whitney U test or Kruskal–Wallis test whereas p-value of categorical variables was calculated by Chi-square test and Fisher’s exact test. Comparison of the two CAC percentiles of total BC patients, left-side and right-side BC patients, and non-BC women was performed by paired t-test. Comparison of %CACinc between left-side and right-side BC patients was performed by Mann–Whitney U test. Regression of %CACinc and clinical factors were executed by linear regression. Spearman correlation was used to calculate the association of %CACinc and dosimetric variables. Logistic regression was used to calculated odds ratio of dosimetric variables which led to %CACinc increase more than 50%. All statistical results were calculated with SAS 9.4 software (SAS Institute, Carry, NC). A two-tailed P value < 0.05 was considered significant.

## Results

**Fig. 1:**
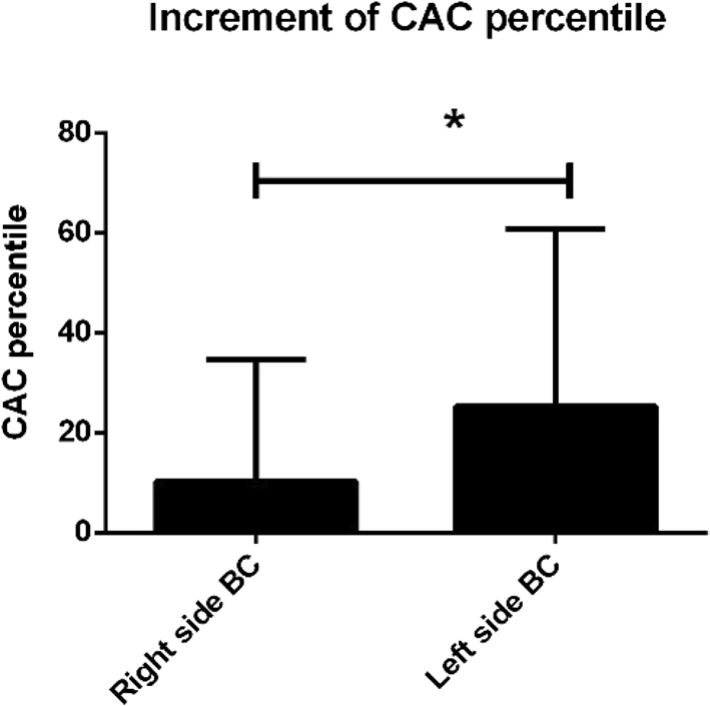
Comparison of increment of CAC percentile (%CACinc) in patients with left-side versus right-side breast cancer

**Fig. 2:**
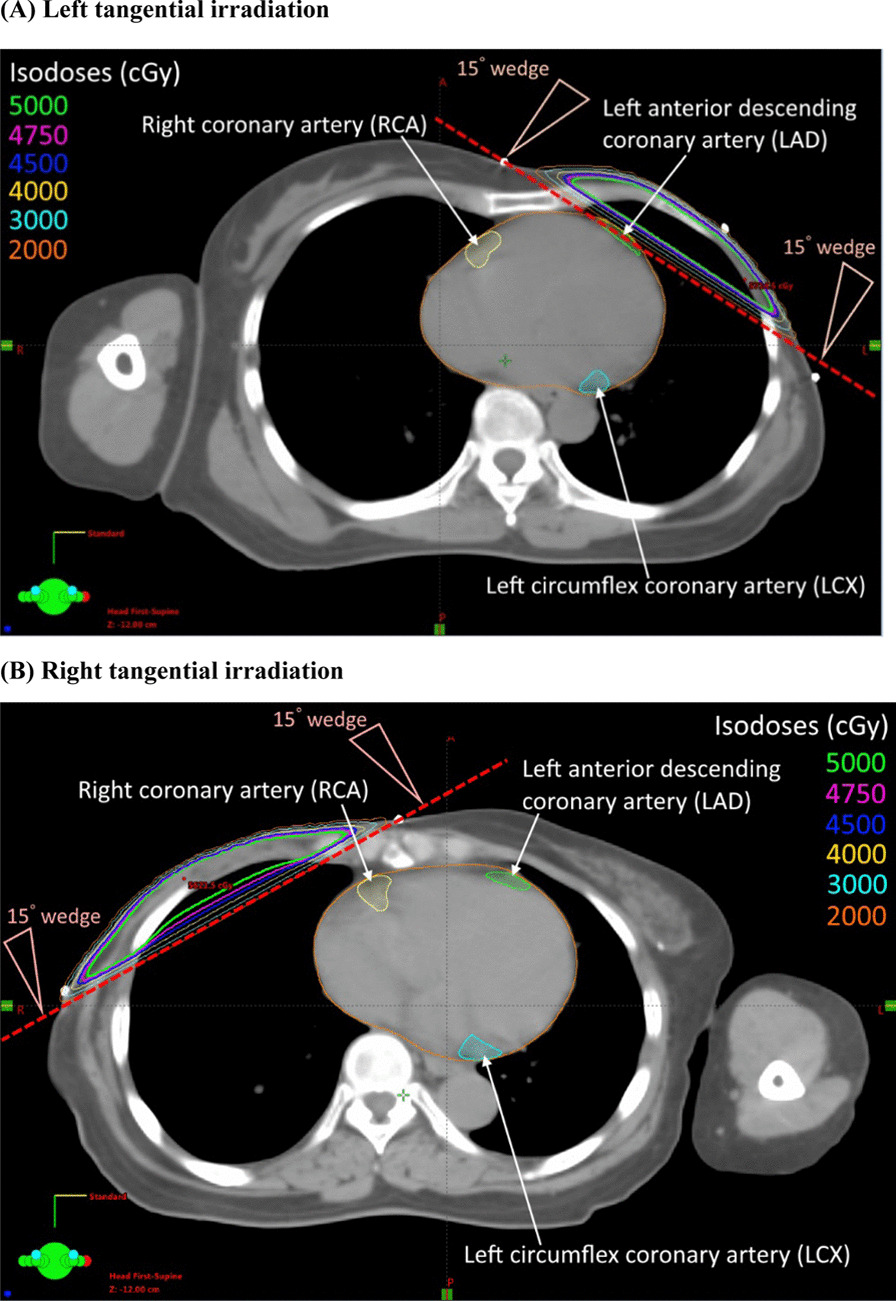
Dose distribution from 6 MV tangential irradiation with 15° wedge in left-side (**a**) and right-side (**b**) breast cancers after mastectomy. The organs at risk (OARs) including the heart, left anterior descending coronary artery (LAD), left circumflex artery (LCX), and right coronary artery (RCA) were outlined

A total of 94 patients were enrolled in this study, including 51 left-side (54.3%) and 43 right-side (45.7%) BC patients. No significant difference of clinical characteristics was presented between left-side and right-side BC patients (Table 1). Before adjuvant RT, 92 out of 94 patients had zero CAC percentiles, and 68 patients still being zero after receiving adjuvant RT. Twenty-six patients had positive %CACinc after adjuvant RT, 19 left-side and 7 right-side BC patients. The mean values of %CACinc from the first to second NCCT were 18.4%, 25.3%, 10.2%, and 2.7% in total, left-side, right-side BC patients, and non-BC women, respectively (Table 2). %CACinc was significantly higher in left-side than right-side BC patients (Fig. 1). The possible factors contributing to clinical characteristics on %CACinc were evaluated in Table 3. %CACinc was increased by 15.13% in left-side BC patients compared with right-side BC patients (p = 0.02), probably because parts of the heart were close to or in the left tangential irradiation fields (Fig. 2). The dosimetric variables between two sides BC patients were analyzed, and results showed that MHD, maximum heart dose, heart V25, mean LAD dose, maximum LAD dose, mean LCX dose, maximum LCX dose, and mean RCA dose were significantly different between two groups (Table 4). The correlations between dosimetric variables were calculated in Table 5, suggesting that MHD, maximum heart dose, mean LAD dose were negatively correlated among dosimetric parameters in right-side BC patients. Besides, mean RCA dose was negatively correlated among dosimetric parameters in right-side BC patients and the overall BC population. We further divided all BC patients into two groups: %CACinc ≥ 50% group and %CACinc < 50% group, and calculated the risks of dosimetric variables which led to increasing more than 50% CAC percentile. After adjusting the laterality of disease, the maximum heart dose did not increase the risks of %CACinc (aOR of 1.015), but the mean RCA dose significantly decreased the risks of enhancing the %CACinc with an aOR of 0.47 (p = 0.039) (Table 6).

**Table 1:** Baseline characteristic of breast cancer patient population

Characteristic	Total	Breast cancer		p value
		Left-side BC	Right-side BC	
Number of patients	94	51 (54.3%)	43 (45.7%)	
Age at 1st NCCT (years)	53 (45–78)	52 (45–74)	55 (45–78)	0.461
Age at 2nd NCCT (years)	60 (48–80)	60 (48–80)	62 (48–79)	0.371
NCCT interval (years)	6.9 (1.0–12.6)	6.8 (1.1–12.6)	7.3 (1.0–11.7)	0.664
Pathological stage				0.292
0	2 (2.1%)	0 (0.0%)	2 (4.7%)	
I	13 (13.8%)	9 (17.6%)	4 (9.3%)	
II	28 (29.8%)	15 (29.4%)	13 (30.2%)	
III	47 (50.0%)	26 (51.0%)	21 (48.8%)	
IV	4 (4.3%)	1 (2.0%)	3 (7.0%)	
Surgery				0.952
BCS	32 (34.0%)	18 (35.3%)	14 (32.6%)	
Mastectomy	62 (66.0%)	33 (64.7%)	29 (67.4%)	
Chemotherapy				0.882
Yes	77 (81.9%)	41 (80.4%)	36 (83.7%)	
No	17 (18.1%)	10 (19.6%)	7 (16.3%)	
Anthracycline or Taxane regimen	0.699			
Yes	76 (80.9%)	40 (78.4%)	36 (83.7%)	
No	18 (19.1%)	11 (21.6%)	7 (16.3%)	
Hormone therapy				0.623
Yes	71 (75.5%)	37 (72.5%)	34 (79.1%)	
No	23 (24.5%)	14 (27.5%)	9 (20.9%)	
Targeted therapy (Trastuzumab)				0.468
Yes	5 (5.3%)	4 (7.8%)	1 (2.3%)	
No	89 (94.7%)	47 (92.2%)	42 (97.7%)	
BMI (overweight)				0.705
≧ 24	49 (52.1%)	28 (54.9%)	21 (48.8%)	
< 24	45 (47.9%)	23 (45.1%)	22 (51.2%)	
BMI (obese)				0.169
≧ 27	25 (26.6%)	17 (33.3%)	8 (18.6%)	
< 27	69 (73.4%)	34 (66.7%)	35 (81.4%)	
Diabetes				0.838
Yes	15 (16.0%)	8 (15.7%)	7 (16.3%)	
No	79 (84.0%)	43 (84.3%)	36 (83.7%)	
Hypertension				0.180
Yes	20 (21.3%)	14 (27.5%)	6 (14.0%)	
No	74 (78.7%)	37 (72.5%)	37 (86.0%)	
Hyperlipidemia				0.142
Yes	13 (13.8%)	10 (19.6%)	3 (7.0%)	
No	81 (86.2%)	41 (80.4%)	40 (93.0%)	

*BC* breast cancer, *NCCT* noncontrast computed tomography, *BCS* breast-conserving surgery, *BMI* body mass index

**Table 2:** Comparison of CAC percentiles between the first and second NCCT in all participants of this study

CAC percentiles	Total BC (n = 94)	Left-side BC (n = 51)	Right-side BC (n = 43)	Non-BC (n = 47)
No increment (zero vs. zero)	68	32	36	30
Positive increment	26	19	7	13
Zero vs. non-zero	24	18	6	1
Non-zero vs. non-zero	2	1	1	12
Positive increment ≥ 50%	22	17	5	1
Mean %CACinc value	18.4%	25.3%	10.2%	2.7%
p value	** < 0.0001**	** < 0.0001**	**0.0096**	0.1551

*CAC* coronary artery calcium, *NCCT* noncontrast computed tomography, *BC* breast cancer, *n* number of patients, *%CACinc* increment of CAC percentiles

Significant difference with p value < 0.05 were shown in bold

**Table 3:** Possible determinants affecting increment of CAC percentiles in breast cancer patients

Variables	β (95% CI)	p value
Laterality of disease		
Right-side BC	Ref	Ref
Left-side BC	**15.13 (2.37, 27.87)**	**0.020**
Pathology stage		
I	Ref	Ref
II	− 10.46 (− 30.09–9.17)	0.296
III	− 5.71 (− 23.90–12.49)	0.539
IV	− 17.07 (− 51.09–17.46)	0.333
Pathology stage		
0 vs. I vs. II	Ref	Ref
III vs. IV	0.215 (− 12.90, 13.33)	0.974
Surgery		
Mastectomy	Ref	Ref
BCS	− 3.08 (− 16.85, 10.70)	0.659
Chemotherapy		
No	Ref	Ref
Yes	− 6.94 (− 23.86, 9.98)	0.417
Hormone therapy		
No	Ref	Ref
Yes	− 2.10 (− 17.29, 13.10)	0.785
Targeted therapy		
No	Ref	Ref
Yes	− 6.10 (− 35.19, 22.10)	0.678
Overweight		
BMI < 24	Ref	Ref
BMI ≧ 24	− 6.19 (− 19.21, 6.83)	0.347
Obese		
BMI < 27	Ref	Ref
BMI ≧ 27	1.18 (− 13.61, 15.97)	0.874
Diabetes		
No	Ref	Ref
Yes	13.99 (− 3.62, 31.60)	0.118
Hypertension		
No	Ref	Ref
Yes	1.62 (− 14.34, 17.59)	0.841
Hyperlipidemia		
No	Ref	Ref
Yes	− 1.59 (− 20.52, 17.34)	0.867
Total irradiation dose (Gy)	− 0.37 (− 1.32, 0.58)	0.439

*CAC* coronary artery calcium, *BC* breast cancer, *BCS* breast-conserving surgery, *BMI* body mass index, *Gy* gray

Significant p value was in bold

**Table 4:** Dosimetric parameters in left-side and right-side tangential irradiation

Dosimetric parameter	Population (n = 75)		p value
	Left-side BC (n = 41) Mean (± SD)	Right-side BC (n = 34) Mean (± SD)	
Total irradiation dose (Gy)	57.6 (± 6.8)	58.2 (± 6.6)	0.711
Mean heart dose (Gy)	4.2 (± 1.4)	1.2 (± 0.6)	** < 0.001**
Maximum heart dose (Gy)	51.6 (± 7.9)	5.1 (± 1.9)	** < 0.001**
Heart V25 (%)	4.0 (± 2.6)	0	** < 0.001**
Heart volume (cm^3^)	572.9 (± 123.3)	573.5 (± 76.3)	0.982
Mean LAD dose (Gy)	19.6 (± 9.7)	1.2 (± 0.7)	** < 0.001**
Maximum LAD dose (Gy)	50.7 (± 7.2)	2.1 (± 1.2)	** < 0.001**
LAD volume (cm^3^)	10.0 (± 4.7)	10.2 (± 4.1)	0.856
Mean LCX dose (Gy)	1.5 (± 0.7)	0.4 (± 0.2)	** < 0.001**
Maximum LCX dose (Gy)	2.5 (± 2.1)	0.7 (± 0.3)	** < 0.001**
Mean RCA dose (Gy)	2.1 (± 0.8)	2.7 (± 0.9)	** < 0.01**
Maximum RCA dose (Gy)	5.1 (± 7.5)	4.6 (± 1.8)	0.703

*BC* breast cancer, *n* number of patients, *SD* standard deviation, *Gy* gray, *LAD* left anterior descending artery, *LCX* left circumflex artery, *RCA* right coronary artery

Significant p value was in bold

**Table 5:** Correlation between dosimetric parameters in left-side and right-side tangential irradiation and increment of CAC percentiles

	Overall BC (n = 75)		Left-side BC (n = 41)		Right-side BC (n = 34)	
	Correlation	p value	Correlation	p value	Correlation	p value
Mean heart dose (Gy)	0.021	0.855	− 0.187	0.241	** − 0.44**	**0.009**
Maximum heart dose (Gy)	0.191	0.101	0.09	0.576	** − 0.375**	**0.029**
Heart V25 (%)	0.068	0.565	− 0.124	0.439	0	NA
Mean LAD dose (Gy)	0.144	0.215	0.004	0.977	** − 0.34**	**0.049**
Maximum LAD dose (Gy)	0.199	0.088	0.106	0.510	–0.301	0.080
Mean LCX dose (Gy)	0.106	0.366	− 0.021	0.894	− 0.274	0.116
Maximum LCX dose (Gy)	0.057	0.626	− 0.123	0.442	− 0.275	0.116
Mean RCA dose (Gy)	** − 0.284**	**0.013**	− 0.129	0.423	** − 0.425**	**0.010**

*CAC* coronary artery calcium, *BC* breast cancer, *n* number of patients, *Gy* gray, *LAD* left anterior descending artery, *LCX* left circumflex artery, *RCA* right coronary artery

P-value was calculated by spearman correlation. Significant p value was in bold

**Table 6:** Risks of CAC percentiles increase ≥ 50% between dosimetric parameters

	Univariate		Multivariate	
	Odds ratio	p value	Odds ratio	p value
Mean heart dose (Gy)	1.042 (0.775–1.401)	0.787	0.584 (0.332–1.026)	0.061
Maximum heart dose (Gy)	1.024 (0.998–1.05)	0.070	1.015 (0.92–1.121)	0.760
Mean LAD dose (Gy)	1.033 (0.987–1.081)	0.165	1 (0.999–1.001)	0.998
Mean RCA dose (Gy)	**0.429 (0.215–0.859)**	**0.017**	**0.47 (0.229–0.962)**	**0.039**

*CAC* coronary artery calcium, *Gy* gray, *LAD* left anterior descending artery, *RCA* right coronary artery

Significant *p* value was in bold

## Discussion

In this study, a new concept of the %CACinc was drawn to display the progression of coronary calcium burden in BC patients. After second NCCT, the accelerated CAC burden in BC patients was significantly higher than that in non-BC women. None of the different cardiotoxic therapeutic agents, treatment, and cardiovascular risk factors was observed to influence the increment of %CACinc in BC patients. After adjuvant RT, the left-side BC patients had a higher risk of accelerated coronary calcium burden after adjusting age, race, and gender compared to the healthy MESA cohort. Besides, reducing RCA dose significantly decreased the risks of CAC percentiles increase ≥ 50% after adjusting the disease's laterality.

Our study was the first to introduce the concept of the %CACinc to manifest the progression of coronary calcium burden. The reasons why the age-, race-, and gender-matched CAC percentiles were selected instead of absolute CAC scores as a predictive tool were shown as followed: Firstly, age was a significant risk factor for the acceleration of coronary atherosclerosis. The more aged is, the higher probability of non-zero coronary calcium score is. For example, the estimated probability of a non-zero coronary calcium score for a Chinese woman was 16% in 50 while 55% in 70 [25]. If the age confounder is not corrected, there might be a problem of over or under-estimate of the effect from other risk factors on the progression of coronary calcium burden. Secondly, race/ethnicity was also a confounder for the predictive value of coronary calcium burden due to different lifestyles among races causing different exposures to cardiovascular risk factors, such as more dietary consumption, less physical activity, higher BMI, or more current or former smokers. Therefore, using age-, race-, gender-matched CAC percentiles by comparison with the MESA cohort instead of using absolute CAC scores would, theoretically, be better reflect the real impact of treatment or other cardiovascular risk factors on the increment of coronary calcium burden in BC patients.

Our result indicated that left-side BC patients had a higher risk of accelerated coronary calcium burden after adjuvant RT. It remained debatable for the influence of the laterality of BC on the CAC level [26, 27]. Nevertheless, radiation exposure to cardiac structures is the only significant difference between left- and right-side BCs, and radiation appears to be an independent risk factor of arteriosclerosis [9]. The risk of cardiac toxicity due to adjuvant RT could begin within a few years after treatment and may continue for more than 15 years. Recent studies showed that a significant decrease in cardiac and left coronary artery doses is likely to reduce long-term side effects [28]. Previous epidemiological studies on post-radiotherapy cardiotoxicity showed that the MHD doses are typically described as those received by the entire heart, and it may thus be a dose criterion for RT-induced cardiotoxicity [29, 30]. In a large population-based case–control study published by Darby et al. [1], an increased risk of major coronary events was linearly correlated with MHD by 7.4% per Gy. Another study for Fannish and Swedish subjects announced an elevated risk of heart disease was correlated with MHD by 4% per Gy [31]. However, our data failed to demonstrate a significant association between MHD and %CACinc. Jacob et al. [32] reported that MHD is not enough to predict with confidence individual patient dose to the left ventricle and LAD, and considering the distribution of doses within these cardiac substructures rather than just the MHD is necessary. Conclusively, there is still no clear consensus on the dose constraints for the heart and coronary arteries in BC patients receiving adjuvant RT. In the era of CT-based planning system, whenever possible, the integration of the coronary artery anatomy into RT planning is encouraged, especially in left-side BC irradiation.

In this study, there is an ambiguous relation between accelerated CAC burden and coronary radiation doses. MHD, maximum heart dose, mean LAD dose, and mean RCA dose showed a significant difference between left-side and right-side adjuvant RT and negatively correlated with each other. However, the increase of MHD, maximum heart dose, and mean LAD dose cannot significantly reflect the higher risk of %CACinc. Besides, the multivariate analysis showed that reducing RCA dose significantly decreased the risks of CAC percentiles increase more than 50% after adjusting the disease's laterality. It might be due to the anatomical position of the RCA. The opening and the mesial part of the RCA are closer to the thoracic cavity than the opening and the proximal portion of the LAD, which might lead to more dosage disturbance of adjuvant RT to RCA, either in left-side or right-side RT. The mean RCA dose might also be an alternative predictor for increment CAC burden in BC patients after adjuvant RT. Further studies are warranted to confirm this thought.

### Limitation

There are several limitations in this study. Firstly, the NCCT scan of thorax was not routinely applied as an initial staging or a follow-up exam for BC patients, thus limited this study’s populations. Some pre-treatment images to determine the baseline CAC values were obtained from simulation CT scans for adjuvant RT treatment planning. During simulation CT scan acquisition, patients were instructed to breathe freely, and no ECG triggering was utilized. These compromised the image quality by allowing cardiac motion, high noise levels, and partial volume effect [33, 34]. However, several studies demonstrated the feasibility of CAC quantification from non-triggered CT scans [24, 34–37] and showed good concordance between gated and non-gated CAC scoring [24, 35]. Using the non-triggered planning CT scans to evaluate pre-treatment CAC values can help reduce radiation exposure and medical costs compared to additional CT scans, and be quickly drawn for clinical routine. Secondly, the patients in the study were treated with a conventional technique (tangentially opposed fields) with sequential boost, which might be a limitation in reducing cardiac dose. Several advanced techniques, such as hypofractionated RT with concomitant boost [20], IMRT with simultaneous integrated boost (SIB) [21], or deep inspiration breath-hold (DIBH) [38], help lower cardiac radiation dose. Thirdly, the dosimetric data of 19 of 94 BC patients in this study was missing, which might bias the statistical analysis. Finally, the length of the time interval between two CT scans in a small group of patients was as short as one year, which was likely not long enough to make a valuable assessment of an incremental change in coronary calcium deposition.

## Conclusion

In conclusion, this study revealed that the accelerated CAC burden in BC patients is significantly higher than that in non-BC women, representing that BC might influence the progression of CAC burden. Furthermore, a significantly higher risk of accelerated CAC burden was found in left-side than right-side BC patients after adjuvant RT. Besides, a decrease of the mean RCA dose could reduce more than 50% of the risk of accelerated CAC burden in BC patients. Further large-scale studies are warranted to confirm this finding.

## Data Availability

The datasets analyzed during the current study are available from the corresponding author on reasonable request.

## Abbreviations

CAC: Coronary artery calcium
BC: Breast cancer
RT: Radiotherapy
NCCT: Noncontrast computed tomography
MESA: Multi-Ethnic Study of Atherosclerosis
%CACinc: Increment of CAC percentiles
MHD: Mean heart dose
LAD: Left anterior descending artery
LCX: Left circumflex artery
RCA: Right coronary artery
Gy: Gray
CAD: Coronary artery disease
BCS: Breast-conserving surgery
BMI: Body mass index
TNM: Tumor, node, metastasis
AJCC: American Joint Committee on Cancer
IMRT: Intensity-modulated radiotherapy
OARs: Organs at risk
LMCA: Left main coronary artery
DVHs: Dose-volume histograms
PTV: Planning target volume
ECG: Electrocardiography
HU: Hounsfield unit
3D: Three-dimensional
